# Effect of an Integrated Package of Nutrition Behavior Change Interventions on Infant and Young Child Feeding Practices and Child Growth from Birth to 18 Months: Cohort Evaluation of the Baduta Cluster Randomized Controlled Trial in East Java, Indonesia

**DOI:** 10.3390/nu12123851

**Published:** 2020-12-16

**Authors:** Umi Fahmida, Min Kyaw Htet, Elaine Ferguson, Tran Thanh Do, Annas Buanasita, Christiana Titaley, Ashraful Alam, Aang Sutrisna, Mu Li, Iwan Ariawan, Michael John Dibley

**Affiliations:** 1 Southeast Asian Ministers of Education Organization Regional Center for Food and Nutrition (SEAMEO RECFON), Pusat Kajian Gizi Regional Universitas Indonesia, Jakarta 10430, Indonesia; kyawhtet@gmail.com; 2 School of Public Health, The University of Sydney, Sydney, NSW 2006, Australia; neeloy.alam@sydney.edu.au (A.A.); mu.li@sydney.edu.au (M.L.); michael.dibley@sydney.edu.au (M.J.D.); 3 Sinergi Qalbu Fikri, Depok 16952, Indonesia; 4 Department of Population Health, London School of Hygiene and Tropical Medicine, London WC1E 7HT, UK; elaine.ferguson@lshtm.ac.uk; 5 National Institute of Nutrition, Hanoi 116110, Vietnam; thanhdo.tran@gmail.com; 6 Surabaya Health Polytechnic, Ministry of Health, Surabaya 60282, Indonesia; sannasita74@gmail.com; 7 Faculty of Medicine, Pattimura University, Ambon 97233, Indonesia; christiana_rialine@yahoo.com; 8 GAIN Global Alliance for Improved Nutrition, Jakarta 12950, Indonesia; asutrisna@gainhealth.org; 9 Center for Health Research, Faculty of Public Health, Universitas Indonesia, Depok 16424, Indonesia; i_ariawan@yahoo.com

**Keywords:** cohort, feeding practices, growth, Indonesia, infants and young children, nutrition-sensitive, nutrition-specific

## Abstract

The need for a multisectoral approach to tackle stunting has gained attention in recent years. Baduta project aims to address undernutrition among children during their first 1000 days of life using integrated nutrition-specific and nutrition-sensitive interventions. We undertook this cohort study to evaluate the Baduta project’s effectiveness on growth among children under 2 years of age in two districts (Sidoarjo and Malang Districts) in East Java. Six subdistricts were randomly selected, in which three were from the intervention areas, and three were from the control areas. We recruited 340 pregnant women per treatment group during the third trimester of pregnancy and followed up until 18 months postpartum. The assessment of breastfeeding and complementary feeding practices used standard infant and young child feeding (IYCF) indicators in a tablet-based application. We measured weight and length at birth and every three-months after that. The enumerators met precision and accuracy criteria following an anthropometry standardization procedure. Among the breastfed children, the percentage of children who achieved the minimum dietary diversity score (DDS) and minimum acceptable diet (MAD) was higher for the intervention group than the comparison group across all age groups. The odd ratios were 3.49 (95% CI: 2.2–5.5) and 2.79 (95% CI: 1.7–4.4) for DDS and 3.49 (95% CI: 2.2–5.5) and 2.74 (95% CI: 1.8–5.2) for MAD in the 9–11 month and 16–18-month age groups, respectively. However, there was no significant improvement in growth or reduction in the prevalence of anemia. The intervention was effective in improving the feeding practices of children although it failed to show significant improvement in linear growth of children at 18 months of age.

## 1. Introduction

An estimated 150 million children are stunted worldwide despite progress to improve children’s nutritional status in recent decades [1]. Indonesia is a middle-income country; however, the prevalence of stunting remains one of the highest in the world [2]. The short term and long term consequences of stunting are well recognized, and the most prominent effect is on the developmental outcome of children [3]. The prevalence of stunting among under-five children in Indonesia was stagnant at 36–37% between 2007 and 2013 [4]. Chronic inadequate intakes of energy and nutrients and repeated infections are well-known factors for childhood undernutrition in Indonesia. But other factors also contribute [5] and should be considered when developing programs to reduce stunting. Therefore, the need for a multisectoral approach to tackle stunting has gained attention in recent years.

According to the World Health Organization (WHO) conceptual framework, household and family factors, such as inadequate complementary feeding, inadequate breastfeeding practices, and infections are proximal factors that often overlap and interact with each other to compromise child growth and development [6]. Two recent reviews of stunting determinants in Indonesia indicate that programs to reduce childhood stunting will need to address the poor quality of water, sanitation and hygiene, and increase exclusive breastfeeding practices, household socioeconomic status, and maternal education. Premature birth, short birth length, and low maternal height were also important factors associated with stunting [7]. These reviews suggest a need for a multisectoral approach to reduce stunting in Indonesia.

The Baduta program was launched in Indonesia in 2014 with support from the Global Alliance for Improved Nutrition (GAIN) [8]. Baduta is an Indonesian word that means a child under two years of age. The program aims to address undernutrition among children during the first 1000 days of life using a package of interventions. The program integrated nutrition-specific and nutrition-sensitive interventions as detailed elsewhere [8]. In brief, the program consists of a package of health systems strengthening and behavior change interventions targeting mothers and children, including nutrition-sensitive and nutrition-specific interventions which aim to promote good water, sanitation and hygiene practices (WASH) and complementary feeding practices. These interventions were integrated into the existing health system and implemented by local and international non-government organizations (NGOs), such as Save the Children and district/village health staff.

This study aims to assess the effectiveness of the Baduta program in improving IYCF practices, growth and anemia of children under 2 years of age. The study was registered at Registry for International Development Impact Evaluations-RIDIE STUDY-ID-55ad3b0f60c57.

## 2. Materials and Methods

### 2.1. Study Design and Study Population

The study was a cluster randomized cohort trial conducted in Sidoarjo District and Malang District, East Java. Six subdistricts representing both urban and rural areas were randomly assigned to either intervention or comparison groups. Pregnant women were recruited during the third trimester of pregnancy and followed-up until 18 months postpartum, as described in detail elsewhere [8]. This cohort trial is one of three components evaluating the Baduta program, which also included a study using repeated cross-sectional surveys to assess program impact and a process evaluation.

### 2.2. Sampling

We randomly selected six subdistricts from the 12 subdistricts included in the cross-sectional study. These 12 subdistricts were selected using constrained randomization to ensure a balanced distribution of covariates in the treatment and comparison group subdistrict clusters [8]. The six subdistricts in this cohort study included four subdistricts in Malang (Turen, Tumpang, Gondanglegi, and Jabung) and two subdistricts in Sidoarjo (Sidoarjo and Prambon). We established a sampling frame of pregnant women in their third trimester from all 105 villages in the selected subdistricts from midwives and health volunteers’ client lists. Recruitment started with those women, who had the highest gestational age, and we obtained the required sample size of women from 92 of the 105 villages across all six subdistricts. Recruitment was done over a four-month period from March to July 2015.

### 2.3. Eligibility Criteria

Women in their third trimester (seventh month of pregnancy onwards) based on the last day of menstruation were eligible to join the study. The exclusion criteria at recruitment were (1) severe anemia (Hb < 7 g/dL by hemocue), (2) suffering from any known chronic illness, (3) planning to deliver the baby and remain outside the study area for more than seven days and (4) intending to migrate outside the subdistrict within the next two years. After the child was born, the exclusion criteria were any visible congenital disease or multiple births (e.g., twins).

### 2.4. Sample Size

We calculated a required sample size of 340 mother/infant pairs per group to detect a difference in mean length-for-age Z score between the experimental and control groups at the end of the follow-up of 0.35 Z score. This calculation set the significance level at 5% and assumed 80% power, a design effect of 1.5, and loss to follow-up of 25%.

### 2.5. Intervention

The Baduta program was implemented by several organizations under the coordination of GAIN and the Indonesian Ministry of Health and is described in detail elsewhere [8]. In brief, it includes five components, as summarized below.

#### 2.5.1. Health System Strengthening

1. Baby-Friendly Hospital Initiative (BFHI): Save the Children implemented this program in the district/municipal hospitals and puskesmas [9].
2. Training of village midwives, health workers, and posyandu cadres: Save the Children used the Indonesian Ministry of Health adaptation of the WHO/UNICEF Community IYC Feeding Counselling Package [10] for this training program.

#### 2.5.2. Behavior Change Intervention

3. “Emo-Demos” intervention package: GAIN developed this intervention, which used 12 participative demonstrative activities, based on the “Evo-Eco” theory of psychological and environmental determinants of behavior [11]. Emo-Demos included topics on nutrition during pregnancy (three activities), breastfeeding (three activities), care during pregnancy (one activity), complementary feeding issues (four activities), and handwashing (one activity). Village midwives, posyandu cadres, and village facilitators were trained to deliver Emo-Demos during monthly classes for pregnant women and child growth monitoring events.
4. National television (TV) campaign: It included four high-quality spots with messages on nutrition during pregnancy, breastfeeding, and complementary feeding issues (two spots). The TV spots were aired on five national TV channels. Village facilitators also showed TV spots using tablets during posyandu meetings to pregnant mothers and “street visits”.
5. Promotion of Nazava water filters: They are a low-cost and effective method to purify water, avoiding the need to boil it. GAIN partnered with Nazava’s rural sales network in the Malang and Sidoarjo districts and trained resellers who market the filters to provide educational sessions on water treatment and handwashing. The initial implementation of these five interventions was staggered (Table 1).

### 2.6. Ethics and Informed Consent

The Faculty of Public Health, Universitas Indonesia (323/H2.F10/PPM.00.02/2016), and the Human Research Ethics Committee of the University of Sydney, Australia (Protocol number: 2015/115) granted ethical approval for the study. We gave a written information sheet to pregnant mothers, and we explained the background and objectives of the study carefully to the women. We only invited women to participate in the study if they provided written informed consent.

### 2.7. Data Collection

All data were captured electronically by trained enumerators. The interviewers recorded information on structured, error detecting forms on tablets using the CommCare system from Dimagi and dispatched directly to a server to clean and merge. The field supervisors and the data manager monitored the data quality. At baseline, we administered a questionnaire to collect social, economic, and demographic data. It was based on the Indonesian Demographic and Health Survey and the National Socioeconomic Survey (SUSENAS) and included an inventory of household assets to construct a wealth index [12]. We measured household food security status with the US Household Food Security/Hunger Survey Module (US-FSSM) adapted for Indonesia [13].

### 2.8. Child Feeding Practices

A standard questionnaire on breastfeeding and complementary feeding practices [14] was administered soon after birth and every month in the first six months and then at three monthly intervals until 18 months. We also collected information about the mothers’ intentions to breastfeed their infants during the pregnancy and their breastfeeding self-efficacy at birth and one, three, and six months postpartum.

### 2.9. Dietary Data

Dietary data were collected electronically using a four-pass 24 h dietary recall (24-HR) when the child was 6–8 months, 9–11 months, and 16–18 months of age. For each target group and data collection period, 24-HRs were proportionately collected on all days of the week to account for any day of the week effects on dietary intakes. In subsamples of children, we collected nonconsecutive day repeat 24-HRs (*n* = 40 per intervention group per data collection period) to adjust nutrient intake distributions for intrasubject variability when estimating the percentage at risk of inadequate intakes [15]. Portion sizes were weighed using food scales or estimated using food photographs. The findings on dietary intake of children were reported earlier [16].

### 2.10. Anthropometry

Trained research assistants (RA) collected duplicate measurements of weight, recumbent length (young children), and height (mothers) using standardized methods and equipment. Weight was measured to the nearest 0.1 g accuracy using SECA 874 digital weighing scales (SECA GmbH & Co.KG, Humburg, Germany), and height was measured using ShorrBoard to the nearest 0.1 cm (Weigh and Measure, LLC, Olney, Maryland, USA). The anthropometrist took a third measurement if the difference between the first two measurements exceeded a predetermined allowable limit. The tablets were also programmed to warn when the recorded values were extreme to alert the anthropometrist of a potential error and the need for remeasurement [17]. We determined the technical error of measurement and systematic error (bias) from a standardization procedure for each anthropometrist before collecting the anthropometric data [17]. If required, we gave them further anthropometric training. We measured the mid-upper-arm-circumference (MUAC) of the pregnant women at baseline (third trimester) and the mothers’ weight and height at the third and 18th month postpartum. We measured the infants’ weight and length at the first and third month, and after that every three months until aged 18 months. We recorded the child’s weight and height from their delivery record for the mothers who gave birth at health facilities. If the baby was delivered at home, we measured the anthropometry within 72 h after birth. We calculated Z-scores for infant length-for-age (LAZ), weight-for-age (WAZ), and weight-for-length (WLZ), using the 2006 WHO Child Growth Standard. We defined stunting, wasting, and underweight as less than-2.00 Z-scores of LAZ, WLZ, and WAZ, respectively. The field team referred children with very low weight-for-height (Z score < −3) to the local puskesmas to assess and treat their severe acute malnutrition. We retained these children in the study.

### 2.11. Anemia

Hemoglobin of children was assessed by Hemocue using finger-prick blood at the age of 18 months. We defined anemia as hemoglobin of less than 11.0 g/dL. We used a hemoanalyzer to assess the hemoglobin of the mother in the third trimester of pregnancy.

### 2.12. Study Outcomes

The primary outcome variable was the child’s LAZ. The secondary outcome variables were the child’s breastfeeding practices, complementary feeding practices and morbidity.

### 2.13. Data Analysis

Data analysis was done by intention to treat and adjusted for the cluster randomization [18]. We analyzed these outcome variables after considering the repeated measurements using separate mixed models. We used linear mixed models for continuous outcomes (e.g., LAZ) and generalized linear mixed models for noncontinuous outcomes (e.g., mixed logistic models for binary outcomes such as percentage exclusively breastfed). Models included intervention/comparison group as a fixed effect, infants as a random effect to account for repeated measurements, and community-cluster as a random effect to account for cluster effects. The reference category, for the analyses, was the comparison group. We used STATA V.15 (StataCorp. 2017. Stata Statistical Software: Release 15. College Station, TX: Stata Corp LP) for all analyses.

## 3. Results

**Figure 1:**
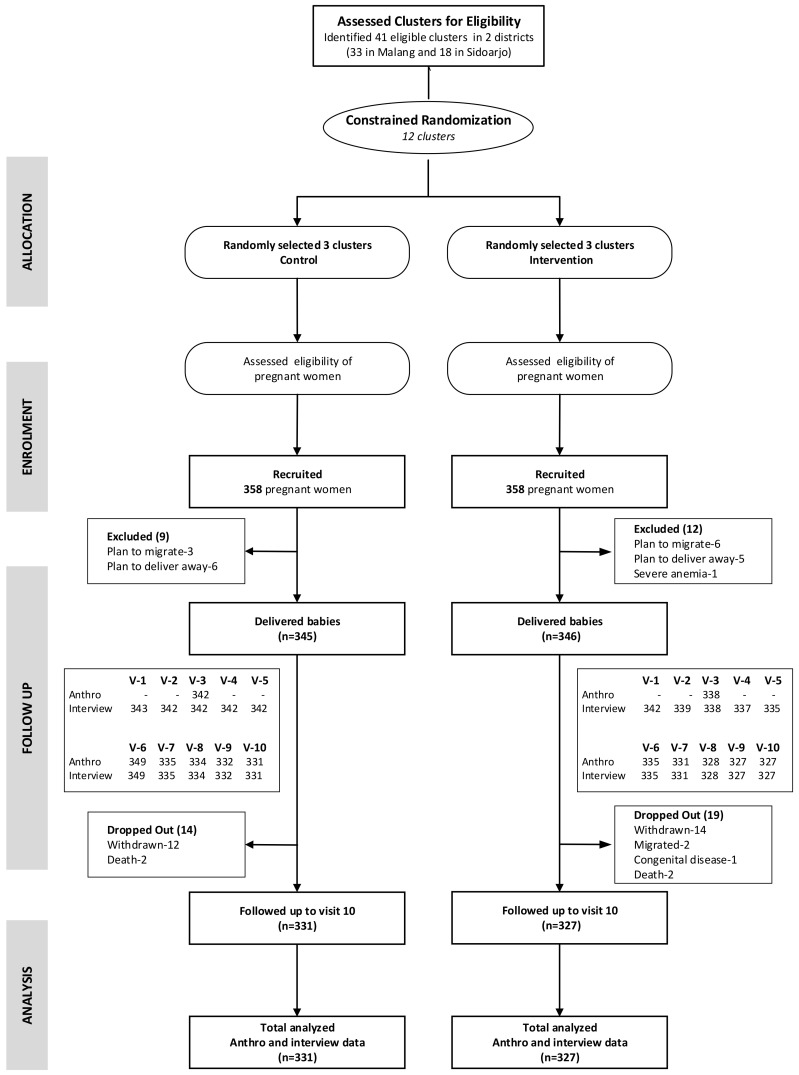
Participant flowchart of the study.

In this cohort evaluation, we successfully followed a total of 658 mother-child pairs up to 18 months after delivery (Figure 1).

Table 2 presents the household-level characteristics of cohort subjects at baseline by treatment group. It shows the treatment groups are balanced for sociodemographic characteristics, except for the type of toilet (i.e., more private toilet in the intervention group).

The baseline individual-level maternal characteristics were well-balanced by the treatment group (Table 3). Around half of the mothers in both groups were anemic. The majority of women in the treatment and comparison groups had a normal mid-upper-arm circumference, and approximately 17% had a low MUAC. The rate of low birth weight was low (Table 3). Overall, there were no significant differences between the groups for birth weight, infant gender, or immunization record (Table 4).

There were no significant inter-group differences in early infant feeding practices (Table 4). Mothers breastfed nearly all children (99%) and similarly fed colostrum (~95%). Close to half of the infants also received prelacteal food, typically formula milk (84%). Mothers of over half of the children had initiated breastfeeding within one hour of birth in both treatment groups. There were no significant intergroup differences in the percentage of children fed prelacteal feeds, breastfed within one hour of birth, exclusively breastfed from birth to six months of age, or still breastfed at one year of age (Table 4).

Among breastfed children, the percentage who achieved a minimum dietary diversity score and a minimum acceptable diet was significantly higher at all age groups in the intervention versus the comparison group (Table 5). These differences were not significant for nonbreastfed 16–18-month-old children. The median dietary diversity scores in both treatment groups increased from two food groups at six to eight months of age to four food groups at 16–18 months of age (data not shown). There were no differences in either the episodes or days with illness over the past two weeks, for cough and fever, at any of the three-monthly morbidity visits (total of six visits throughout the study). However, for diarrhea the figure was lower in the intervention than comparison group (median (5th–95th)) i.e., 0 (0–1) vs. 0 (0–2), *p* = 0.045 for episode and 2 (1–7) vs. 3 (1–10), *p* = 0.056 for days with diarrhea.

There were no statistically significant differences between treatment groups comparing LAZ, hemoglobin, and percentages of children who were stunted or anemic (Table 6). The prevalence of child stunting increased from approximately 5% at three months of age to around 25% at 15 months and 18 months of age. Over half of the children were anemic at 18 months of age (Table 6).

## 4. Discussion

The findings from our evaluation of this project demonstrate that the package of integrated interventions had a positive impact on child feeding practices. However, the program did not improve child growth or reduce the prevalence of anemia over its initial 12 months of implementation (starting from the time when intervention covered at least 50% of the target group). Among the breastfed children, the percentage of children who achieved the minimum dietary diversity and minimum acceptable diet was higher for the intervention than the comparison group across all age groups. This finding is encouraging because it shows an apparent improvement in one of the immediate causes of childhood undernutrition [5].

Several factors contributed to the lack of impact of the Baduta program on child growth and anemia. Firstly, there was no difference in breastfeeding practices between intervention and comparison groups. The Indonesian National Basic Health survey (Riskesdas) found that the exclusive breastfeeding rate decreased from 54% in 2016 to 46.7% in 2017 and 37.3% in 2018. The Baduta project’s findings revealed that exclusive breastfeeding was even lower than the current national data, i.e., only 24% of women exclusively breastfed their children up to six months. This figure is concerning since exclusive breastfeeding in the first six months of life is critical for adequate child growth and development. There are also consequences later in life for children, not exclusively breastfed [19]. In the Baduta project, coverage, for the breastfeeding component of the intervention was low (<20%), which meant intergroup differences in exclusive breastfeeding rates were not sufficient to positively impact linear growth at an early age. This finding suggests the need for a longer duration of exposure or intensity of intervention to improve exclusive breastfeeding rates. Successful implementation of the Baby Friendly Hospital Initiative (BFHI) and Emo-Demo activities would have significantly improved breastfeeding practices. A recent study in India found an integrated nutrition and health program (Infant and Young Child Nutrition—INHP II) effectively improved breastfeeding and linear growth for children under six months. But there was no significant increase in linear growth at the end of the 18-month intervention [20]. The authors proposed that a predominantly cereal-based diet and lack of animal source food at an older age might be the reason why there was no significant impact on child growth after six months. The INHP II program aimed to strengthen the quality and coverage of a package of health and nutrition services during pregnancy until the first two years of age. Both INHP II and the Baduta program aim to increase linear growth through improving IYCF practices. In the INHP II program, significant improvement in linear growth up to five months was achieved, possibly due to better breastfeeding practices in the intervention group, which was not achieved in the Baduta program.

Besides the Baduta program’s Emo-Demo approach, TV advertisements were also used as a communication strategy. The Baduta program broadcasted TV advertisements throughout the province in both intervention and control areas; therefore, mothers in control subdistricts also potentially viewed them. This may have partly contributed to the lack of significant differences between intervention and comparison groups in the breastfeeding indicators, including prelacteal feeding, exclusive breastfeeding, and continued breastfeeding. The most intensive broadcasting of TV ads on exclusive breastfeeding was in the second quarter of 2016. Nonetheless, the findings suggest they did not have sufficient influence to improve mothers’ existing breastfeeding practices. The most intensive broadcasting of TV ads on complementary feeding was in the fourth quarter of 2016, which coincided when most cohort study subjects were 5–11 months and allowed more time to influence their mothers’ feeding practices.

In the Baduta project, we observed a positive impact on dietary practices, although it failed to improve growth and anemia significantly. The percentage of children who achieved a minimum dietary diversity score (DDS ≥ 4) and a minimum acceptable diet was significantly higher among the children in the intervention group (Table 5). More importantly, the consumption of animal-source food (meat/fish/poultry and egg, ASF) was significantly higher among the children in the intervention group. Consumption of ASF, an important source of iron and zinc intake, is an important predictor for child growth. The Human Nutrition Collaborative Research Support Program in Kenya, Mexico, and Egypt found a significant association between ASF consumption and young children’s linear growth. [21]. The quality of diet, especially ASF consumption, is recognized as an important determinant of children’s cognitive outcomes starting from newborns where the maternal intake of ASF was a significant predictor for newborn habituation behavior [22]. The effect can be long-lasting. Earlier studies among Kenyan children showed that ASF intake at a young age was a significant predictor of cognitive performance even at five years [23]. Therefore, although we did not observe any impact of those dietary practices on linear growth, we expect children in the intervention group of the Baduta project could benefit at a later age. Earlier studies have shown that animal source food consumption promotes linear growth and is associated with reduced stunting [24,25]. Zaharia et al. demonstrated the role of ASFs in linear growth. They found that past ASF intake was more strongly correlated with linear growth than the current intake, suggesting that increased intakes of ASFs identified in the intervention group were more significant with longer study duration [26]. A recent study in Nepalese children under five years old showed that increasing the dietary diversity by one food group was associated with only a 0.09 cm (95% CI: 0.00, 0.17 cm) increase in height [27], which is a small change even though statistically significant. Consistent with this finding, even though the percentage of children meeting the minimum dietary diversity score was higher in the intervention group, it was only associated with a 0.1 and 0.05 higher LAZ score at age 9–11 months and 16–18 months, respectively.

Repeated infection is a common reason for chronic undernutrition among children. The Baduta program addressed this risk factor by including a handwashing activity in the Emo-Demo session and providing Nazava water filters to households in the intervention area. The aim was to reduce the number of episodes of common infections, such as diarrhea. The intervention package was weakly associated with reduced illness. The number of times children had diarrhea between the three-monthly visits and the number of days of diarrhea reported in the two weeks before each follow-up visit were borderline significantly lower in the intervention group. Based on data from seven cohort studies, Richard et al. showed that diarrhea, was only associated with a loss in height potential after an accumulation of episodes over the first 24 months of life [28]. The authors concluded that even though a single diarrhea episode during childhood has a small but measurable negative effect on linear growth, it is compensated by catch-up growth when there is adequate illness-free time. Therefore, the difference in diarrhea observed between intervention and control groups was unlikely to be a significant contributor to the nutritional status of children in the Baduta project.

Finally, it is important to note that the intervention components, which included Emo-Demo activities, counseling by midwives, and village facilitators’ visit, started at different time points throughout the intervention. Given the complexity of the program’s operations and the difference in the starting time of each program component, it is necessary to study program exposure as it evolved as well as to identify program component (s) and pathway, which is/are significant in improving feeding practices and growth outcomes.

There are limitations in the study, especially the timing of introducing different interventions, resulted in different duration of exposure and failed to constitute a comprehensive package. Consequently, the impact on linear growth and anemia was limited and failed to show a significant effect. Previous evidence suggested that positive effect on weight gain is not always accompanied by significant effect on linear growth [29]. While the effect on growth may take time, the impact on biochemical status is more likely to appear sooner. Therefore, it would have been relevant to assess micronutrient status, especially iron and zinc status. Finally, in the intervention group, a social desirability bias might have influenced a mother’s recall of her child’s dietary intakes, given the intervention’s focus on improved complementary feeding practices. The low percentage of six-month-old intervention children achieving the minimum DDS and MAD, however, suggests a social desirability bias was less likely, although it cannot be discounted. Furthermore, while we observed a positive impact on feeding practices, the breastfeeding practices remained poor, especially the rate of exclusive breastfeeding. Therefore, overall, the results suggest the communication interventions need to be intensified, for example, using innovative technologies such as mobile apps to educate and closely follow up mothers to encourage appropriate infant and young child feeding practices [30].

## 5. Conclusions

In conclusion, this cohort evaluation of the Baduta program failed to find a significant impact on child growth, however, it did demonstrate an improvement in infant feeding practices. Earlier implementation and higher coverage of the “IYCF Practices Pathway” activities than what was achieved at the time of this study might have resulted in higher exclusive breastfeeding rates and greater improvements in dietary diversity from six months of age onwards in the intervention than the comparison group, which may have positively affected growth. An integrated package of interventions is expected to improve infant and young children feeding practices and child growth, but it should be delivered early with adequate coverage to ensure adequate exposure to the intervention which can lead to positive outcome on child growth.

## Figures and Tables

**Table 1:** Percent coverage of the intervention throughout study period from third trimester pregnancy until 18 month postdelivery (summarized version).

	2015												2016											
	Q1			Q2			Q3			Q4			Q1			Q2			Q3			Q4		
	J	F	M	A	M	J	J	A	S	O	N	D	J	F	M	A	M	J	J	A	S	O	N	D
Child age (month)			−3			0	1	2	3	4	5	6			9			12			15			18
Intervention																								
a. Iron Intake	0%			1%			1%			19%			43%			59%			63%			67%		
b. Exclusive Breastfeeding	11%			16%			20%			34%			57%			64%			67%			69%		
c. IYCF Practices	0%			1%			5%			29%			42%			61%			69%			74%		
d. HWTS and Handwashing	1%			3%			6%			10%			18%			26%			58%			68%		

a Knowledge—adoption Iron Intake Pathway for Pregnant Women; average of six indicators: (1) % posyandus monitoring of iron folic acid intake pregnancy; (2) % of villages with trained village midwife on Pregnant Women Class; (3) % of villages to deliver three Emo-Demo sessions in pregnant woman class; (4) % of pregnant women to attend at least one iron intake Emo-Demo; (5) % of the pregnant woman shown ATIKA TVC by village facilitator on a tablet to promote consumption of liver, egg and fish (in Bahasa Indonesia Ati, Telur, IKAn or ATIKA); (6) % of mothers with child <2 y and pregnant women registered to SMS Bunda; b Knowledge—adoption Exclusive Breastfeeding Pathway; average of six indicators: (1) % of health facilities with maternity services implement BFHI; (2) % of villages with village midwives who received breast feeding Counseling Training; (3) % of posyandus delivering three Emo-Demo sessions for EBF; (4) % of mothers with child attending at least one Emo-Demo session on EBF; (5) % of mothers with <2 y child watching EBF TVC on a tablet from village facilitator; (6) % of mothers with <2 y child and pregnant women registered with SMS Bunda; c Knowledge—adoption IYCF Practices Pathway; average of six indicators: (1) % posyandus with at least one community health worker received infant and young child feeding (IYCF) counseling training; (2) % of villages with village midwife having received IYCF counseling training; (3) % of posyandus to deliver four Emo-Demo sessions for IYCF; (4) % of mothers with <2 y child attending at least one IYCF Emo-Demo; (5) % of mothers with <2 y child shown IYCF TVC by village facilitator on a tablet; (6) % of mothers with <2 y child and pregnant women registered with SMS Bunda; d Knowledge—adoption HWTS and Handwashing Pathway; average of seven indicators: (1) % villages with at least one free distribution of Nazava water filter; (2) % villages with at least one education session for safe water treatment and storage; (3) % villages with at least one Nazava water filter sale; (4) % of posyandus to deliver an Emo-Demo session on handwashing; (5) % of mothers with <2 y child attending an Emo-Demo session on handwashing; (6) % of mothers with <2 y child shown handwashing TVC by village facilitator on a tablet; (7) % of mothers with <2 y child and pregnant women registered with SMS Bunda.

**Table 2:** Household characteristics of cohort mothers by treatment group.

Characteristics	Intervention (*n* = 346)		Comparison (*n* = 345)		*p*-Value ^1^
	*n*	%	*n*	%	
District of household					
Sidoarjo	121	33.2	119	32.6	0.991
Malang	243	66.8	246	67.4	
Source of drinking water					
Piped water	32	8.8	40	11.0	
Well pump	43	11.8	71	19.5	
Protected well	124	34.1	54	14.8	
Protected spring	16	4.4	80	21.9	
Water cart/truck	44	12.1	1	0.3	0.266
Refilled water	32	8.8	51	14.0	
Branded mineral water	67	18.4	51	14.0	
Nonprotected source	1	0.3	8	2.2	
Other	5	1.4	9	2.5	
Water treatment before drinking					
Boiled	267	99.6	271	100.0	0.315
Filtered/chlorinated/other	1	0.4	0	0.0	
Type of toilet/latrine facility					
Private	338	92.9	292	80.0	
Public	3	0.8	13	3.6	0.015
Shared	19	5.2	20	5.5	
No toilet	4	1.1	40	11.0	
Housing characteristics					
Electricity					
Government	364	100.0	364	99.7	0.408
Other	0	0.0	1	0.3	
Flooring material					
Marble	259	71.2	246	67.4	
Tiles	13	3.6	5	1.4	
Brick	86	23.6	79	21.6	0.429
Bamboo	0	0.0	1	0.3	
Dirt/Soil	6	1.7	34	9.3	
Wall material					
Plastered wall	352	96.7	330	90.4	0.211
Non-plastered wall	12	3.3	35	9.6	
Roof material					
Roof tile	341	93.7	356	97.5	0.383
Other	23	6.3	9	2.5	
Cooking fuel					
Polluting fuel	40	11.0	76	20.8	0.424
Nonpolluting fuel	324	89.0	289	79.2	
Household assets					
Television	358	98.4	351	96.2	0.301
AC	16	4.4	6	1.6	0.390
Water heater	2	0.6	2	0.6	0.999
LPG 12 kg or more	12	3.3	8	2.2	0.703
Refrigerator	199	54.7	176	48.2	0.826
b. Means of transport					
Bicycle	240	65.9	239	65.5	0.980
Motor	355	97.5	348	95.3	0.307
Boat without engine	0	0.0	3	0.8	0.249
Motorboat	0	0.0	1	0.3	0.396
Car	25	6.9	28	7.7	0.831
Household wealth index quintiles at baseline					
Lowest	40	11.0	110	30.1	
Second	101	27.8	84	23.0	
Middle	58	15.9	52	14.3	0.498
Fourth	99	27.2	54	14.8	
Highest	66	18.1	65	17.8	
Food Security ^2^					
Level of food security					
Food secure	288	79.1	256	70.1	
Food insecure without hunger	71	19.5	97	26.6	0.428
Food insecure with hunger	5	1.4	12	3.3	

^1^ Chi-square to test for independence to assess any significant difference between treatment groups. *p* < 0.05 indicates statistical significance; ^2^ household food security status measured by the US-Household Food Security/Hunger Survey Module (US-FSSM).

**Table 3:** Baseline characteristics of cohort mothers by treatment group.

Characteristics	Intervention (*n* = 346)		Comparison (*n* = 345)		*p*-Value ^1^
	*n*	%	*n*	%	
Mother’s age in years					
<15	1	0.3	1	0.3	
15–19	23	6.3	38	10.4	
20–29	199	54.7	165	45.2	0.263
30–39	134	36.8	145	39.7	
≥40	7	1.9	16	4.4	
Mother’s education					
No school/incomplete primary	5	1.4	10	2.7	
Completed primary school	65	17.9	107	29.3	
Completed junior high school	108	29.7	124	34.0	0.466
Completed senior high school	148	40.7	108	29.5	
Diploma/University	38	10.4	16	4.4	
Mother’s occupation					
Housewife	276	75.8	300	82.2	
Government/Private	31	8.5	13	3.6	
Entrepreneur	28	7.7	21	5.8	0.433
Factory worker	17	4.7	18	4.9	
Other	12	3.3	13	3.6	
Pregnancy history					
Total number of pregnancies					
First pregnancy	131	36.0	133	36.4	
Second pregnancy	155	42.6	141	38.6	0.519
Third pregnancy	51	14.0	64	17.5	
Fourth and more pregnancy	27	7.4	27	7.4	
Number of miscarriages					
Never	354	97.3	354	97.0	0.371
Once or more	10	2.7	11	3.0	
Gestational age at the time of enrollment					
Pregnancy in days (Mean, SD)	243	25	234	26	0.062
Anemia status					
No anemia (Hb ≥ 11.0)	148	43.5	175	50.3	
Mild anemia (10.0 ≤ Hb ≤ 10.9)	94	27.7	110	31.6	0.088
Moderate anemia (7.0 ≤ Hb ≤ 9.9)	98	28.8	63	18.1	
Hb (mean, SD)	10.6	1.31	10.9	1.26	0.145
Mid-upper-arm circumference					
Malnourished (<23 cm)	61	16.8	63	17.3	0.916
Normal (≥23 cm)	303	83.2	302	82.7	
MUAC (mean, SD)	26.3	3.77	26.1	3.41	0.812

^1^ Chi-square to test for independence to assess any significant difference between treatment groups. *p* < 0.05 indicates statistical significance.

**Table 4:** Child characteristics and breastfeeding practices by treatment group.

Characteristics	Intervention (*n* = 346)		Comparison (*n* = 345)		*p*-Value ^1^
	*n*	%	*n*	%	
Girls	182	52.6	172	49.9	0.409
Low birth weight (birth weight < 2500 g)	12	3.5	20	5.8	0.073
Breastfeeding					
Ever breastfed	340	99.4	338	99.1	0.751
Initiation of breastfeeding after delivery					
Immediately (within one hour)	158	46.2	180	52.8	
Within hours (1 to 24 h)	18	5.3	19	5.6	0.705
Within days	163	47.7	141	41.3	
Don’t know	3	0.9	1	0.3	
Colostrum feeding, i.e., gave the first yellowish breastmilk to the baby	322	94.7	321	95.0	0.981
Prelacteal feeding i.e., gave prelacteal food to baby	147	43.0	171	50.1	0.654
Type of prelacteal food					
Formula milk	140	87.0	160	82.5	0.561
Drinking water	7	2.0	18	5.3	0.186
Water with sugar	5	1.5	3	0.9	0.447
Honey	23	6.7	27	7.9	0.866
Banana	3	0.9	18	5.3	0.069
Coconut water	5	1.5	4	1.2	0.885
Other	10	2.9	11	3.2	0.917
Percentage of children exclusively breastfed up to 6 months					
1.0 to 1.9 month	181	52.9	147	43.0	0.542
2.0 to 2.9 month	156	45.6	125	36.5	0.494
3.0 to 3.9 month	131	38.3	111	32.5	0.599
4.0 to 4.9 month	111	32.5	98	28.7	0.708
5.0 to 5.9 month	83	24.3	67	19.6	0.536
Continued breastfeeding at one year	257	79.6	247	76.2	0.744
Have immunization	308	94.2	327	97.6	0.224

^1^ Chi-square to test for independence to assess any significant difference between treatment groups. *p* < 0.05 indicates statistical significance.

**Table 5:** Complementary feeding practices, by treatment group.

Characteristics	Intervention (*n* = 346)		Comparison (*n* = 345)		Adjusted Odds Ratio ^1^			*p*-Value
	*n*	%	*n*	%	OR	LCI	UCI	
Percentage of children achieving minimum meal frequency ^2^								
6–8 m, breastfed	269	97.1	277	97.9	0.73	0.25	2.13	0.562
9–11 m, breastfed	263	97.4	254	93.7	2.51	1.03	6.17	0.044
16–18 m, breastfed	240	98.4	231	97.9	1.30	0.34	4.90	0.700
16–18 m, nonbreastfed	75	100	89	98.9				
Percentage of children achieving minimum dietary diversity score ^3^								
6–8 m, breastfed	42	15.2	27	9.5	1.69	1.01	2.83	0.045
9–11 m, breastfed	139	51.5	62	22.9	3.49	2.20	5.54	0.000
16–18 m, breastfed	185	75.8	124	52.5	2.79	1.75	4.45	0.000
16–18 m, nonbreastfed	59	78.7	62	68.9	1.46	0.38	5.62	0.578
Percentage of children achieving minimum acceptable diet ^4^								
6–8 m, breastfed	41	14.8	27	9.5	1.64	0.98	2.76	0.059
9–11 m, breastfed	139	51.5	62	22.9	3.49	2.20	5.54	0.000
16–18 m, breastfed	184	75.4	124	52.5	2.74	1.78	4.23	0.000
16–18 m, nonbreastfed	49	65.3	43	47.8	1.80	0.42	7.67	0.428

^1^ Odds ratio—adjusted for cluster sampling. *p* < 0.05 indicates statistical significance.(the comparison group was the reference category); ^2^ consuming a diet with minimum meal frequency during the previous day; ^3^ consuming at least four out of seven food groups; ^4^ minimum acceptable diet for breastfed children = diets with the minimum dietary diversity (≥ four food groups) and the minimum meal frequency during the previous day. Minimum acceptable diet for non-breastfed children = children who received at least two milk feeds and had diets with minimum dietary diversity not including milk feeds (≥ four food groups) and minimum meal frequency during the previous day.

**Table 6:** Nutritional status of cohort children, by age and treatment groups.

**Characteristics**	**Intervention**		**Comparison**		**Mean Difference**			***p*-Value ^2^**
					**Adjusted ^1^**	**LCI**	**UCI**	
**Length-for-age Z scores ^4^**	**Mean**	**SD**	**Mean**	**SD**				
Month 3	−0.40	0.94	−0.59	0.92	0.15	−0.16	0.45	0.337
Month 6	−0.62	0.96	−0.73	0.98	0.08	−0.23	0.38	0.612
Month 9	−0.81	0.98	−0.95	0.97	0.10	−0.20	0.41	0.511
Month 12	−1.10	1.01	−1.24	0.94	0.10	−0.21	0.40	0.523
Month 15	−1.30	1.01	−1.37	0.92	0.04	−0.27	0.34	0.818
Month 18	−1.38	0.97	−1.47	0.94	0.05	−0.26	0.36	0.747
Hemoglobin								
Hemoglobin (g/dL)	10.48	1.29	10.39	1.40	0.06	−0.04	0.17	0.186
**Stunting ^5^**	***n***	**%**	***n***	**%**	**Odds Ratios**			***p*-Value**
					**Adjusted ^3^**	**LCI**	**UCI**	
Month 3	11	3.3	23	6.7	0.50	0.20	1.20	0.12
Month 6	31	9.3	30	8.9	1.06	0.52	2.18	0.87
Month 9	42	12.7	41	12.2	1.05	0.54	2.06	0.88
Month 12	60	18.3	61	18.3	1.03	0.55	1.94	0.92
Month 15	82	25.1	84	25.3	1.04	0.57	1.89	0.91
Month 18	83	25.4	91	27.5	0.97	0.53	1.76	0.92
**Anemia status at 18 months**								
Normal (> 11 g/dL)	107	41.0	120	46.7				
Mild (10.0–10.9 g/dL)	75	28.7	87	33.9	1.24	0.82	1.86	0.31
Moderate (7.0–9.9 g/dL)	79	30.3	50	19.5				

^1^ Adjusted RR was calculated using multilevel Poisson regression, adjusted for wealth index (the comparison group was the reference category); ^2^ *p*-value for chi-square test for differences between intervention and comparison groups adjusted for the complex sample design and adjusted for household wealth index using random-effect logistic or linear regression models, *p* < 0.05 indicates statistical significance; ^3^ adjusted mean difference was calculated using a multilevel linear regression adjusted for wealth index; ^4^ excludes flagged values with Z score > +6.00 or <−6.00; ^5^ stunting = length-for-age < –2 Z score using 2006 WHO growth standards.
